# Use of a Geographic Information System to create treatment groups for group-randomized community trials: The Minnesota Heart Health Program

**DOI:** 10.1186/s13063-019-3284-9

**Published:** 2019-03-28

**Authors:** Brittany Krzyzanowski, Steven M. Manson, Milton Mickey Eder, Len Kne, Niki Oldenburg, Kevin Peterson, Alan T. Hirsch, Russell V. Luepker, Sue Duval

**Affiliations:** 1 Department of Geography, Environment & Society, University of Minnesota, Minneapolis, MN USA; 2 Cardiovascular Division and Lillehei Heart Institute, University of Minnesota Medical School, 420 Delaware Street SE, MMC 508, Minneapolis, MN 55455 USA; 3 Department of Family Medicine and Community Health, University of Minnesota Medical School, Minneapolis, MN, USA; 4 U-Spatial, Office of Vice President for Research, University of Minnesota, Minneapolis, MN USA; 5 Division of Epidemiology and Community Health, School of Public Health, University of Minnesota, Minneapolis, MN, USA

**Keywords:** Group-randomized trials, Community intervention methods, Public health interventions, Geographic information system (GIS)

## Abstract

**Background:**

Group-randomized trials of communities often rely on the convenience of pre-existing administrative divisions, such as school district boundaries or census entities, to divide the study area into intervention and control sites. However, these boundaries may include substantial heterogeneity between regions, introducing unmeasured confounding variables. This challenge can be addressed by the creation of exchangeable intervention and control territories that are equally weighted by pertinent socio-demographic characteristics. The present study used territory design software as a novel approach to partitioning study areas for The Minnesota Heart Health Program’s “Ask about Aspirin” Initiative.

**Methods:**

Twenty-four territories were created to be similar in terms of age, sex, and educational attainment, as factors known to modify aspirin use. To promote ease of intervention administration, the shape and spread of the territories were controlled. Means of the variables used in balancing the territories were assessed as well as other factors that were not used in the balancing process.

**Results:**

The analysis demonstrated that demographic characteristics did not differ significantly between the intervention and control territories created by the territory design software.

**Conclusions:**

The creation of exchangeable territories diminishes geographically based impact on outcomes following community interventions in group-randomized trials. The method used to identify comparable geographical units may be applied to a wide range of population-based health intervention trials.

**Trial registration:**

National Institutes of Health (Clinical Trials.gov), Identifier: NCT02607917. Registered on 16 November 2015.

## Background

In an ideal multisite community intervention trial (CIT), intervention and control communities would be exact replicates of one another before random assignment to ensure that the study outcome is most likely due to the impact of the intervention. However, as the ideal set of paired and conveniently defined community units is unobtainable, investigators must be resourceful when identifying practical and comparable geographical research units [1]. Unfortunately, multisite CITs often rely on the convenience of pre-existing administrative divisions (e.g., school districts, census tracts, etc.) to delineate the geographical regions that will be randomized to the intervention and control groups, and these units are not guaranteed to be exchangeable in terms of community characteristics. One way to develop comparable communities is *regionalization* [2, 3], the process of combining smaller geographical units within the geography of interest to create – by convenience – artificial communities that are comparable [4]. Regionalization affords researchers the ability to build experimental communities from underlying data, customizing the population within treatment groups according to pre-selected study parameters.

The present study evaluated the utility of a regionalization tool as a novel approach to create balanced territories for public health and community intervention research (Business Analyst Territorial Design, ESRI, Redlands, CA, USA). *ESRI’s Business Analyst Extension* is one of many existing tools developed for a traditional use in optimizing business and marketing strategies, using consumer demographic datasets that would be applicable to corporate efforts to create regions or zones to “balance” sales, service, and advertising efforts. We hypothesized that such an analytic approach would be equally useful to create optimal territories for dissemination of population-based community health interventions. To the best of our knowledge, this study represents the first use of this territory design tool by a public health intervention study to balance geographic units at baseline.

The Minnesota Heart Health Program’s (MHHP) “Ask about Aspirin” Initiative is aimed at increasing the appropriate use of aspirin for the primary prevention of myocardial infarction (MI) and stroke. The study design is a *group-randomized trial* (GRT), with geographic regions or *territories* serving as the groups or *clusters*. The creation of these exchangeable treatment groups, or *territories*, is the focus of this paper.

## Methods

The randomization units for the “Ask about Aspirin” study are 24 territories in the state of Minnesota. The intervention consists of a health system intervention applied in a crossover fashion to half of the territories in the first 2 years, and to the other half in the subsequent 2 years, against a background of a statewide media campaign. The health system intervention promotes the integration of the 2009 United States Preventive Services Task Force (USPSTF) aspirin recommendations [5] as a part of a health system’s quality improvement (QI) initiative for its primary care clinics. Measurement of the primary outcome, appropriate aspirin use for the primary prevention of cardiovascular disease, is by means of telephone surveys of 100 individuals in each of the 24 study territories.

Within regionalization studies [6], several parameters are defined in order to control the shape, size, and statistical makeup of the territories. The available parameter settings vary among territory design software (some having more settings than others) but, in general, most regionalization software provides investigators with the basic capability to customize the number of regions, their compactness, and to achieve balance between the study areas according to key underlying attributes (e.g., population density) [1]. Other common parameter settings allow investigators to keep territory boundaries from crossing over other boundaries (e.g., county, zip code, or school district boundaries), and integrate seed points (i.e., centers of interest) and drive time or drive distance (to or from points of interest) into the territory design process. In what follows, we describe the parameter settings selected for the present study using ESRI’s Business Analysis Territory Design (https://www.esri.com/library/whitepapers/pdfs/territory-design.pdf).

In this Minnesota state-based GRT, it was known from prior research that aspirin use for primary prevention of cardiovascular disease (CVD) varies by age, sex, and socioeconomic status, commonly measured by educational level and annual income [7]. No current standard geographic units in Minnesota balance these important variables.

The territory partitions were constrained to align with zip codes in order to facilitate the conduction of telephone surveys, which were administered only to households with landline telephones. A list of landline telephone numbers was obtainable by zip code, which could then be aggregated to the territory level. The population-dense seven-county (Anoka, Carver, Dakota, Hennepin, Ramsey, Scott, and Washington counties) Twin Cities’ (Minneapolis and Saint Paul) metropolitan area was excluded as a geographic unit in order to minimize cross-unit health system contamination, because patients within population-dense metropolitan regions are known to receive care across many clinics. Furthermore, it is difficult to separate a public health intervention message by region, but this is especially true for the metropolitan area where the communication market is not easily divisible into segments. Rochester, MN (home of the Mayo Clinic) was also excluded from the study since it is a unique city dominated by the health care industry, thereby precluding an appropriate match with a like region. This restriction limited the study’s ability to generalize to large urban areas; however, the potential contamination (as described in the “Methods” section) was deemed to be unavoidable.

The core goal of regionalization is the aggregation of smaller sub-units into larger, comparable study regions. The territories to be utilized in the Ask About Aspirin study were thus designed to be similar in terms of age, sex, and education. Education was chosen as a proxy for socioeconomic status (rather than use of an income variable), because educational attainment is discrete, easy to measure, and remains relatively stable over adulthood [8]. The territory design software allows for a maximum of five variables to balance territories. Age, sex, and education were combined into four variables: men aged 45–79 years, women aged 55–79 years, men aged 45 years and over with at least some college education, and women aged 45 years and over with at least some college education. Age groups were determined according to the 2009 USPSTF aspirin-use guidelines [5]. The four balancing variables were weighted as follows: 20% for men aged 45–79 years, 20% for women aged 55–79 years, 30% for men over 45 years with at least some college education, and 30% for women aged over 45 years with at least some college education. Educational attainment was weighted more heavily as it is considered to be a more important determinant of aspirin use [9].

To assess the overall quality of the balancing, data were also gathered on other relevant factors including marital status and average household income. Age, sex, marital status, and income data were collected from the Business Analyst-provided demographic dataset constructed from the Census and the American Community Survey (http://www.esri.com/data/esri_data/explore-data). Education data were collated from the 2013 National Historical Geographic Information System (NHGIS) (http://www.nhgis.org) dataset.

In addition to seeking between-unit comparability (as defined by population characteristics), regionalization of spatial data is also concerned with maintaining compact territory shapes [10]. Compactness of a territory is measured with a compactness score (a value of between 0 and 100) whereby higher compactness scores are closer to a perfect circle. Within the present study, both maintaining maximum compactness and balancing demographic characteristics among the territories were important, but a trade-off exists between the two [3, 11]. Perfectly balanced territories that contain similar populations are rarely compact. The key rationale for maintaining compactness is that spatial analysis often assumes that spatially proximate entities are more similar [12] and by compacting territories, the maximum distance from territory edges is minimized and entities within the territory are considered proximate to each other. In this particular study, the primary care clinic intervention required practice facilitators to implement intervention strategies at adjacent clinics within their assigned territories, having compact (rather than elongated) territories helped to ensure that nearby clinics would fall within the same territory. For these reasons, more weight was allocated to compacting territories over balancing the demographic variable criteria. However, setting the territory design software to provide territories with a high compactness score affects the overall balancing of socioeconomic demographics between territories. Despite this, the difference was determined to be negligible in terms of the overall homogeneity observed among the territories in the final output.

Health system- and clinic-based practice interventional effectiveness likely varies according to health system-based and clinic factors, such as administrative and physician leadership, QI focus, and dedication to the use of practice change tools [13]. Thus, it was deemed important, a priori, to assure that study territories contained at least two health systems, and several clinics within those health systems, to avoid confounding by health system.

**Fig. 1:**
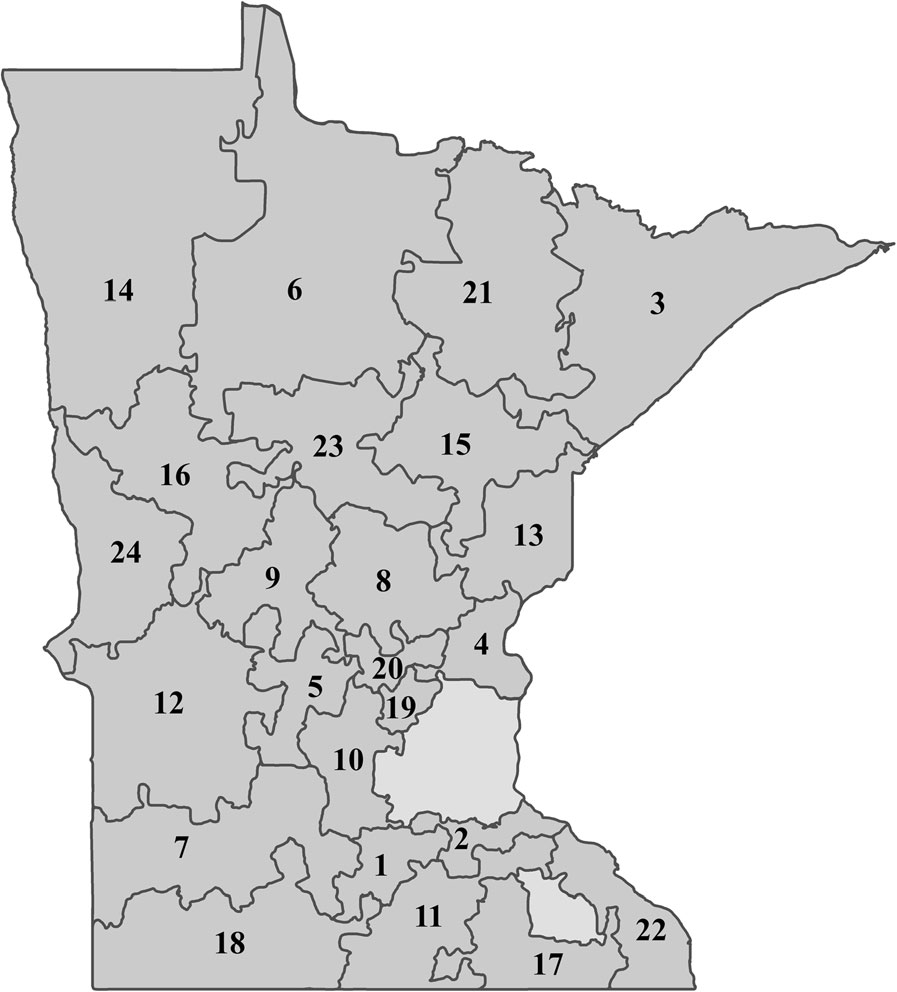
Map of Minnesota showing the 24 geographic territories created for the “Ask About Aspirin” group-randomized trial using Geographic Information Systems (GISs). The Twin Cities’ metropolitan area and Rochester, MN were not included (areas with no associated number)

**Fig. 2:**
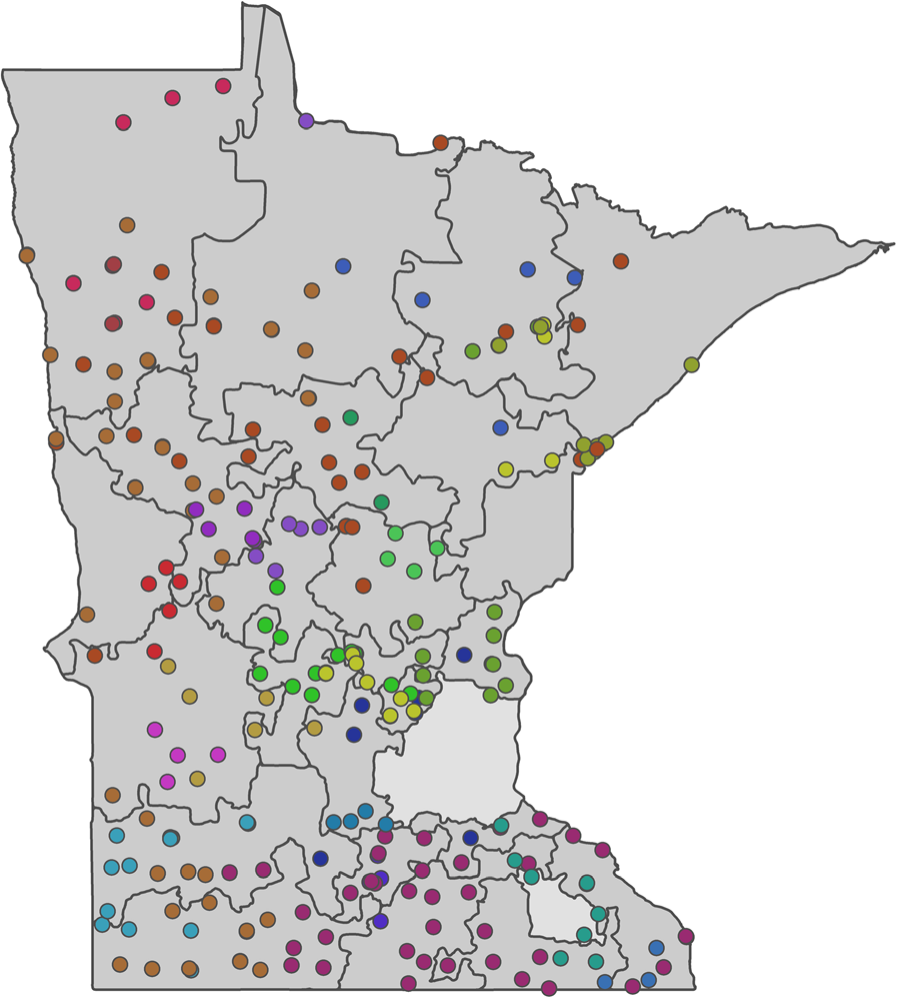
Minnesota map showing the Minnesota Heart Health Program (MHHP) territories with the clinics superimposed, color-coded such that the same color indicates the same health system. Note that at least two different health systems appear in each territory

**Fig. 3:**
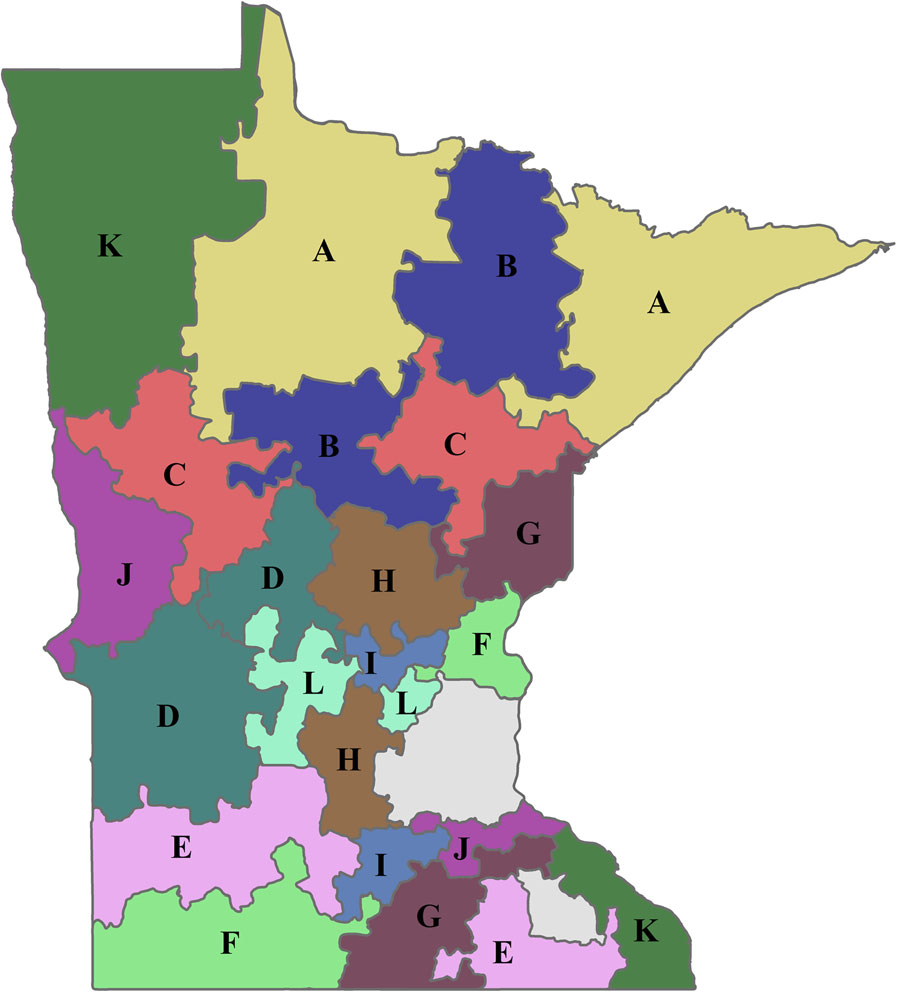
Map showing pair-matched territories. Territories were ranked according to education (see the “Methods” section), and pair-matched. Each member of the pair is shown on the map with the same letter, **a** though **l**

**Fig. 4:**
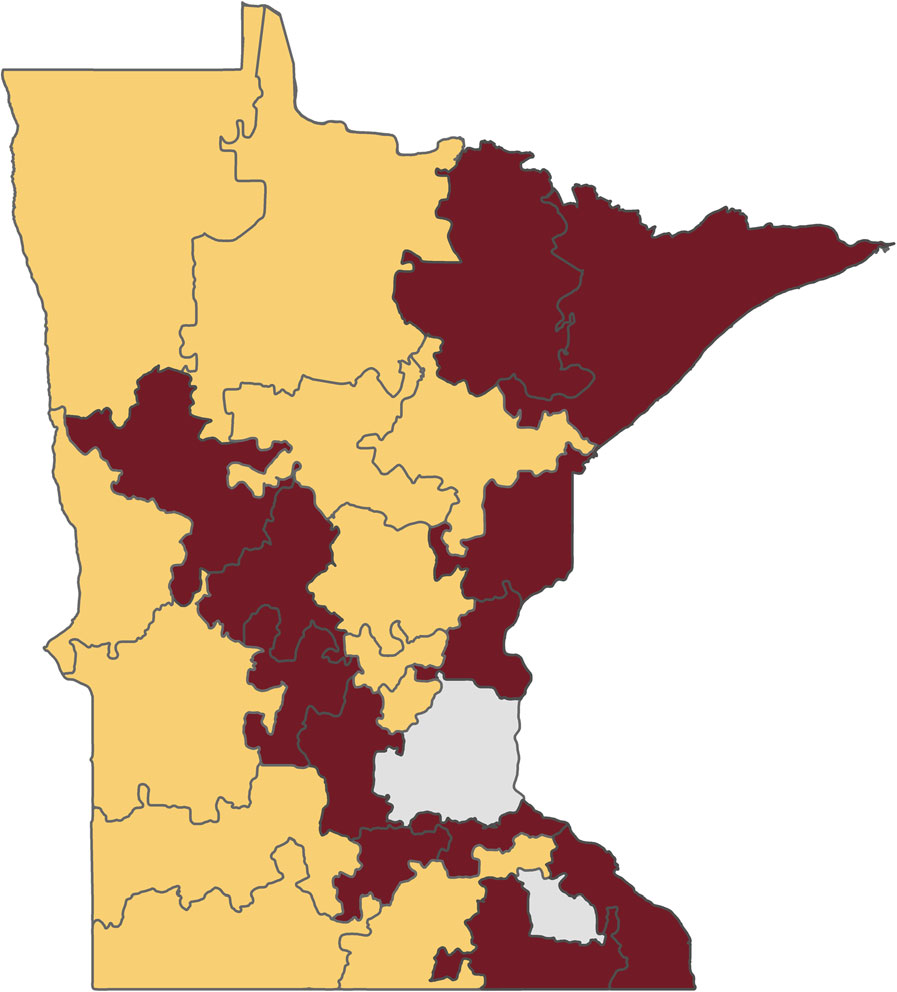
Map of Minnesota Heart Health Program (MHHP) treatment groups. Map shows intervention and control groups, after each pair of territories was randomized to treatment group

In summary, the territories were created via the following steps. The initial territories were produced by setting the number of territories required to 24. These were then balanced by the variables determined to modify aspirin use (age, sex, and education), which were assigned weights according to their known impact on aspirin use. Several iterations of the territory design with different compactness settings were performed until a solution with at least two health systems per territory was produced. A compactness setting of 88% gave the best solution (Fig. 1). Primary care clinic locations were then plotted against the map produced by the software, and assigned colors to denote their corresponding health care system (Fig. 2). Subsequently, the 24 territories were ranked by the percentage of men aged 45–79 years and women aged 55–79 years with at least some college education within each territory (Table 1). Territories were then paired using this ranking variable (Fig. 3, pairs have the same letter), and the paired territories were assigned either “maroon” or “gold” according to a coin toss (Fig. 4). Lastly, a coin toss decided whether the 12 “maroon” territories or the 12 “gold” territories were randomized to the intervention or the control.

**Table 1:** Sociodemographic variables used in balancing territories

Territory	Population	Health systems	Clinics	Age^a^	Education^b^
1	113,130	2	12	29.8%	20.6%
2	101,354	3	7	33.9%	23.0%
3	66,044	2	7	43.7%	32.1%
4	105,208	2	8	36.6%	20.8%
5	122,163	3	10	29.1%	17.7%
6	56,200	4	12	59.0%	36.7%
7	99,234	5	16	37.1%	21.1%
8	108,314	4	8	36.0%	20.5%
9	85,959	4	11	41.3%	23.8%
10	105,068	3	8	35.3%	20.0%
11	100,036	3	13	36.8%	21.7%
12	87,645	5	12	39.7%	24.1%
13	96,459	4	9	35.6%	21.0%
14	92,863	4	21	37.9%	22.1%
15	82,760	4	7	38.8%	25.2%
16	83,813	4	18	41.6%	24.7%
17	98,259	3	10	35.8%	22.0%
18	99,767	4	22	38.3%	21.1%
19	129,107	4	9	28.3%	18.7%
20	115,249	3	9	30.8%	19.5%
21	70,827	5	13	44.7%	29.1%
22	98,525	3	11	35.8%	22.2%
23	63,611	3	9	53.0%	33.8%
24	94,754	3	5	33.8%	23.4%

^a^Prevalence of men aged 45–79 years and women aged 55–79 years

^b^Prevalence of men aged 45–79 years and women aged 55–79 years with at least some college education

Demographic variables used in balancing the territories and those not used in the balancing process are reported as mean (standard deviation (SD)).

## Results

There was no significant difference between intervention and control groups in any of the variables used to balance the territories (Table 2). The mean number of men aged 45–79 years in the intervention territories compared to the control territories was very similar, as was the mean number of women aged 55–79 years. Similarly, there were only very small differences in the mean number of men or women with at least some college education in the intervention territories compared to the control territories.

**Table 2:** Descriptive statistics of the newly formed control and intervention groups. Each group consists of 12 territories

	Intervention	Control
*Variables used in balancing territories*		
# Health systems, range	3–5	2–5
# Clinics, total	144	123
Target age group^a^		
# Women	15,755 (976)	16,129 (949)
# Men	18,827 (1518)	19,190 (1380)
Target education group^b^		
# Women	11,227 (634)	11,236 (474)
# Men	10,259 (468)	10,366 (703)
*Variables not used in balancing territories*		
Sex (%)		
Women	49.5 (0.7)	49.9 (0.4)
Men	50.5 (0.7)	50.1 (0.4)
Median age, years		
Women	43.7 (3.3)	44.1 (4.7)
Men	42.3 (3.5)	43.3 (4.4)
Education level		
# Less than high school	5706 (1335)	5673 (1611)
# High school	37,416 (5497)	36,307 (6613)
# College	19,444 (2485)	18,226 (4969)
# Postgraduate/professional	4556 (1363)	4144 (1359)
Average income ($US)	69,611 (3069)	65,682 (9600)
# Married	47,517 (13,293)	46,298 (17,514)

Values are mean (SD), unless noted otherwise

*#* number

^a^Men aged 45–79 years, women aged 55–79 years

^b^At least some college education

Furthermore, we found that several variables not involved in the balancing process were very similar between treatment groups (Table 2). The groups were well matched in the proportion of men and women, sex-specific median age, and categories of educational attainment. The mean number of married individuals, and average household income between the intervention and control territories were also not significantly different.

## Discussion

The territory design software produced control and intervention territories for the study that were not significantly different from one another in terms of key socioeconomic characteristics which are known to differ in aspirin use for primary prevention of cardiovascular disease.

One of the advantages of the proposed new territory design strategy in support of community-based interventional trials is that it allows investigators to easily create custom-built territories according to pre-selected, scientifically valid parameters that support study goals. Among these parameters are those that can be used in weighting territories by attributes of interest, compacting the geographic shape of the territory, and setting an alignment layer (keeping territory boundaries from crossing over pre-specified boundaries). Although there are other methods to create geographic units, we focused this work only on those with significant implications for public health research.

In spite of the fact that territory design software provides an efficient approach to produce exchangeable study regions, it does not guarantee internal validity [1]. These strategies are still subject to the issues that accompany group-randomized trials (e.g., confounding by unmeasured variables, crossover effects, and loss to follow-up). Territory design software may improve the probability that such bias is minimized.

Another limitation of this method is the potential splitting of pre-existing communities. Within the present study, each territory contained several communities (e.g., neighborhoods, cities), and because the boundaries of the territories were constrained to align with zip codes (and communities do not necessarily align with zip codes), a community could potentially rest between the borders of two or more territories. Larger cities that contain multiple zip codes have the potential to be split over more than one territory, and may be assigned to different treatment groups. This introduces another level of complexity to a study given the mobility of individuals within large communities. For this reason, among others, the present study excluded the seven-county metropolitan area of Minneapolis-Saint Paul. It is also worth noting that policies are implemented based on other geographical units (subnational governments or territorial jurisdictions) and, therefore, do not match up with the created territories. In order to avoid this disconnect, investigators could constrain their regionalization strategy to align with relevant territorial jurisdictions.

The assignment of experimental conditions to territories that are exchangeable in their population characteristics allows for more scientifically robust comparisons. Territory design software can thus be used to enhance the validity of public health research, likely improving operational efficiency at study onset, during the intervention, and upon data analysis. It is important to note that zone design software can enhance the research process for other domains as well. Within the domain of agriculture for instance, growth and allocation research, land use planning, and irrigation strategies are still very much reliant on the use of rectangular partitioning or grids to designate plots [14, 15]. Software exists that can support optimization of these tasks, and currently, such analytic programs are underutilized (or not utilized at all). This is also the case for community intervention research.

## Conclusion

With methodological advantages over existing approaches, territory design software proves to be a useful tool, enhancing the validity of public health research and further saving time and effort in creating study area divisions. The territory design software produced control and intervention territories that were not significantly different from one another in terms of pertinent socioeconomic characteristics, and, thus, minimized potential confounding. Despite this, these types of analytical programs are underutilized within public health research. Future research is warranted to evaluate the advantages of the use of traditional public health methods for territory design vs. the proposed use of geographic mapping software.

## Abbreviations

CIT: Community intervention trial
CVD: Cardiovascular disease
GIS: Geographic Information System
GRT: Group-randomized trial
MHHP: Minnesota Heart Health Program
MI: Myocardial infarction
NHGIS: National Historical Geographic Information System
QI: Quality improvement
SD: Standard deviation
USPSTF: United States Preventive Services Task Force

## References

[1] Folch DC, Spielman SE (2014). Identifying regions based on flexible user-defined constraints. Int J Geogr Inf Sci.

[2] Duque JC, Ramos R, Suriñach J (2007). Supervised regionalization methods: a survey. Int Reg Sci Rev.

[3] Ricca F, Simeone B (2008). Local search algorithms for political districting. Eur J Oper Res.

[4] Openshaw S (1977). A geographical solution to scale and aggregation problems in region-building, partitioning and spatial modelling. Trans Inst Br Geogr.

[5] US Preventive Services Task Force (2009). Aspirin for the prevention of cardiovascular disease: U.S. Preventive Services Task Force Recommendation Statement. Ann Intern Med.

[6] Li W, Goodchild MF, Church R (2013). An efficient measure of compactness for two-dimensional shapes and its application in regionalization problems. Int J Geogr Inf Sci.

[7] Luepker RV, Steffen LM, Duval S, Zantek ND, Zhou X, Hirsch AT. Population trends in aspirin use for cardiovascular disease prevention 1980–2009: The Minnesota Heart Survey. J Am Heart Assoc. 2015;4(12). 10.1161/JAHA.115.002320. 10.1161/JAHA.115.002320 PMC4845283 26702085

[8] Krieger N, Williams DR, Moss NE (1997). Measuring social class in US public health research: concepts, methodologies, and guidelines. Ann Rev Public Health.

[9] Luepker RV, Rosamond WD, Murphy R, Sprafka JM, Folsom AR, McGovern PG, Blackburn H (1993). Socioeconomic status and coronary heart disease risk factor trends: The Minnesota Heart Survey. Circulation.

[10] Wise SM, Haining RP, Ma J, Fischer MM, Getis A (1997). Regionalisation tools for exploratory spatial analysis of health data. Recent developments in spatial analysis: spatial statistics, behavioural modelling, and computational intelligence.

[11] Fan C, Li W, Wolf LJ, Myint SW (2015). A spatiotemporal compactness pattern analysis of congressional districts to assess partisan gerrymandering: a case study with California and North Carolina. Ann Assoc Am Geogr.

[12] Tobler W (1970). A computer movie simulating urban growth in the Detroit region. Econ Geogr.

[13] Fallon LF, Begun JW, Riley WJ. Managing health organizations for quality and performance. Burlington: Jones & Bartlett; 2013.

[14] Xie Y, Runck B, Shekhar S, Kne L, Mulla D, Jordan N, Wiringa P (2017). Collaborative geodesign and spatial optimization for fragmentation-free land allocation. ISPRS Int J Geo Inf.

[15] Bergez JE, Garcia F, Lapasse L (2004). A hierarchical partitioning method for optimizing irrigation strategies. Agric Syst.

